# Biofilm-associated molecular patterns: BAMPs

**DOI:** 10.1128/iai.00304-25

**Published:** 2025-08-21

**Authors:** Peter Østrup Jensen, Morten Rybtke, Tim Tolker-Nielsen

**Affiliations:** 1 Costerton Biofilm Center, Department of Immunology and Microbiology, University of Copenhagen 232207, Copenhagen, Denmark; 2 Department of Clinical Microbiology, Copenhagen University Hospital (Rigshospitalet) 683301, Copenhagen, Denmark; 3 Center for Rheumatology and Spine Diseases, Institute for Inflammation Research, Copenhagen University Hospital (Rigshospitalet) 53167, Copenhagen, Denmark; University of California at Santa Cruz, Santa Cruz, California, USA

**Keywords:** biofilm, infection, BAMPs, innate immune response

## Abstract

Chronic infections involving bacterial biofilms are a major clinical challenge due to the ability of biofilm to resist antimicrobial treatments and host immune responses. The resulting persistent infections are often accompanied by collateral damage mainly executed by activated components of the innate immune system in response to the infectious biofilm. The innate immune system responds to the recognition of pathogen-associated molecular patterns (PAMPs), which are broadly expressed by both planktonic and biofilm-forming bacteria. However, the expression of special PAMPs in association with biofilms remains poorly defined. Here, we review prior studies that provide experimental evidence of the existence of immune-activating molecular patterns that are expressed at immunostimulatory levels in biofilms but not in planktonic bacteria. Accordingly, we introduce the concept of biofilm-associated molecular patterns (BAMPs) as a subset of PAMPs that are expressed in biofilms. Identifying BAMPs and elucidating their role in innate immune activation may inform the development of targeted therapies to reduce collateral tissue damage in biofilm-associated infections.

## INTRODUCTION

**Fig 1:**
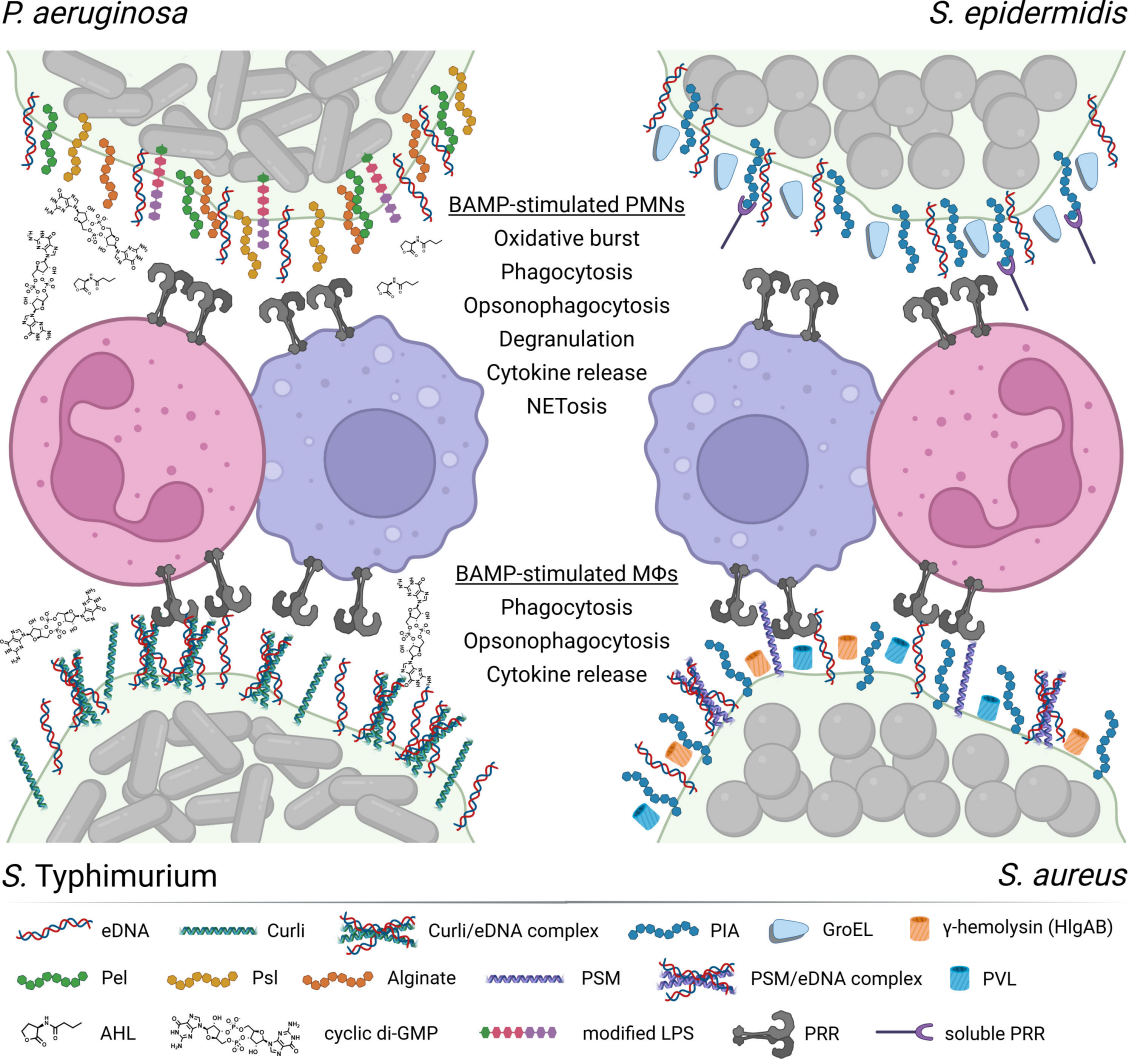
The concept of immunostimulatory BAMPs. Molecular components of the biofilm, e.g., eDNA, polysaccharides, amyloid fibrils, moonlighting proteins, pore-forming toxins, signal molecules, and modified LPS, stimulate innate immune cells. The membrane-bound PRRs involved in detecting the BAMPs range from known Toll-like receptors (e.g., TLR2, TLR4, and TLR9) and c-type lectin receptors (e.g., MR-CTLD4-7 and dectin-2) to unknown receptors yet to be identified. Soluble PRR, e.g., maltose-binding lectin and serum ficolins of the lectin binding pathway, may also play a role in detecting the polysaccharide BAMPs leading to opsonophagocytosis. The primary immune cells are PMNs and macrophages. The two types of immune cells appear together on the figure, but PMNs are usually present before macrophages. See main text for details. (Created in BioRender. Rybtke, M. (2025) https://BioRender.com/5rz63vn).

Biofilms are implicated in a wide range of infections, posing a major challenge to both healthcare and patient outcomes. These structured microbial communities are involved in a variety of infections, e.g., associated with medical implants, wounds, the urinary tract, oral diseases, and respiratory diseases, such as cystic fibrosis (CF) (1, 2). A defining feature of biofilms is the extracellular polymeric substance (EPS) matrix in which the bacteria are embedded (3, 4). This matrix, composed of polysaccharides, proteins, lipids, and extracellular DNA, provides structural integrity to the biofilm and is not produced at high levels by solitary planktonic bacteria. The innate immune system acts as the first line of defense that recognizes and responds to biofilms, attempting to eliminate them before they establish chronic infections. However, the persistent presence of biofilms can lead to prolonged activation of the innate immune response, which may accidentally cause damage to the surrounding tissues (5, 6) and induce microenvironmental changes linked to decreased susceptibility to antibiotic treatment (7, 8). The sustained inflammation stimulated by infectious biofilms can lead to tissue destruction, impaired healing, and contribute to the progression of chronic conditions. The ability of the innate immune system to respond to pathogens relies in part on pattern-recognition receptors (PRRs) recognizing pathogen-associated molecular patterns (PAMPs) (9, 10). The PAMPs are foreign molecular patterns expressed by various forms of intruding microorganisms, but the existence of special subsets of PAMPs expressed exclusively in association with biofilms remains poorly defined. Here, we review prior studies that provide experimental evidence of the existence of immune-activating molecular patterns that are expressed at immunostimulatory levels in biofilms but not in planktonic bacteria. Accordingly, we introduce the concept of biofilm-associated molecular patterns (BAMPs) as a subset of PAMPs that are expressed in biofilms (Fig. 1). BAMPs are expressed within biofilms in a manner that stimulates the innate immune response, whereas planktonic cells do not express BAMPs at levels sufficient to affect the innate immune response. We present evidence that BAMPs are expressed in bacterial biofilms of both Gram-negative and Gram-positive species. We also examine current evidence regarding the identification of components of PRRs involved in the detection of BAMPs. Furthermore, we highlight findings suggesting that BAMPs can be recognized not only by membrane-bound PRRs located on both extracellular and intracellular membranes, but also by soluble PRRs, expanding our understanding of how the innate immune system interacts with biofilms. With this concept of BAMPs, we aim to establish a clearer insight into biofilm-associated molecular patterns and their role in distinguishing biofilm-mediated immune responses from those triggered by planktonic microorganisms, to better understand biofilm-host interactions and chronic infections.

## BAMPs IN *PSEUDOMONAS AERUGINOSA* BIOFILMS

Biofilm formation by *P. aeruginosa* is a significant clinical challenge, e.g., in people with CF (pwCF) with chronic lung infection, and in people suffering from infected chronic wounds (11, 12). The biofilms withstand attacks from antibiotics and the activated host immune response, leading to hyperinflammation that damages the surrounding host tissue. The polymorphonuclear leukocytes (PMNs) act as major contributors to the hyperinflammatory response and the collateral tissue damage during *P. aeruginosa* biofilm infection. Thus, the density of PMNs in CF sputum was more strongly correlated to impaired lung function in pwCF than the density of *P. aeruginosa* (13). PMNs cause damage to the surrounding tissues through their oxidative and proteolytic activities. In CF, the impact of oxidative immune cell activities on lung tissue damage is highlighted by the inverse correlation between oxidized protein in lung specimens and the pulmonary function (14). Similarly, the concentration of proteolytic neutrophil elastase protein in lung specimens is inversely correlated with the lung function (15).

It has long been recognized that PMNs respond with an increased oxidative burst against solitary planktonic *P. aeruginosa* as compared with the oxidative burst against equivalent numbers of attached *P. aeruginosa* biofilm bacteria (16, 17). To detect activation of the oxidative burst of PMNs by biofilms, Rybtke et al. employed a modification of luminol-enhanced chemiluminescence (18, 19), which is a sensitive method for quantitation of PMN activation according to the production of intra- and extracellular reactive oxygen species (ROS) resulting from the oxidative burst in response to stimulation (20). As expected, a stronger oxidative response was raised by PMNs against solitary planktonic *P. aeruginosa* than against *P. aeruginosa* biofilm. Nevertheless, *P. aeruginosa* biofilm still elicited a strong oxidative burst by the responding PMNs (18).

Being a major part of the innate immune response, the PMNs employ PRRs to detect and respond to specific molecular patterns (9, 10). To assess the ability of PMNs to recognize BAMPs, isolated human PMNs were exposed to *P. aeruginosa* biofilms that were genetically engineered to express the biofilm matrix components Psl, Pel, and alginate at varying levels (18). The PMNs produced greatly increased levels of ROS specifically in response to biofilm with simultaneous overexpression of alginate and Psl, indicating the ability of PMNs to recognize an alginate- and Psl-rich biofilm matrix. To ensure that the PMN response reflected the direct stimulation of PMNs by the biofilm with modified matrix, serum was avoided (18). Otherwise, the complement system in serum may have led to opsonization of *P. aeruginosa* biofilm with C3 fragments engaging complement receptors on the PMNs, leading to confounding the response against matrix exopolysaccharide, since stimulatory opsonization by C3 mainly occurs by recognizing LPS (21). Furthermore, serum may contain antibodies against bacterial antigens, and these antibodies might have been introducing confounding PMN responses by opsonizing the biofilm and stimulating the Fc-receptors of PMNs. Having excluded confounding contributions by the humoral components of the immune system, the results demonstrated that PMNs are able to respond by increased production of ROS when exposed to *P. aeruginosa* biofilm specifically with alginate- and Psl-rich matrix. This is suggestive of PMNs expressing PRRs capable of recognizing specific components of the matrix EPS of *P. aeruginosa* biofilm (18). It cannot be ruled out if the biofilms had shed free Pel, Psl, and alginate, but incubation of PMNs with purified Pel or Psl did not induce a PMN response as evidenced by the absence of an oxidative burst (22). Likewise, incubation of PMNs with purified alginate failed to induce an oxidative burst by PMNs (23, 24). This indicates that the simultaneous overexpression of alginate and Psl in biofilm enhances biofilm-mediated immunostimulatory properties, leading to an increased oxidative burst in the responding PMNs. Since Psl and alginate are produced only in minute amounts by solitary planktonic *P. aeruginosa* (25), it is likely that only biofilm-growing *P. aeruginosa* can express these matrix EPS at levels sufficient to trigger an extra oxidative burst in the responding PMNs. Overexpression of matrix EPS is a hallmark of mucoid *P. aeruginosa* biofilm, which is a major pathologic feature in chronic lung infection in pwCF. In mucoid *P. aeruginosa* biofilms, alginate is overexpressed and Psl and Pel also serve as essential scaffolding components of the matrix (26). Thus, we propose to consider simultaneous overexpression of alginate and Psl as a BAMP. The effect of the genetically manipulated expression of Psl and alginate on the oxidative burst by PMNs induced by solitary planktonic bacteria was, however, not evaluated (18). As recently demonstrated, however, PMNs were crucial for survival only in mice with intra-tracheal infections caused by EPS-producing *P. aeruginosa* but not in those infected with *P. aeruginosa* lacking EPS production (27). In addition to the immunostimulatory properties of overexpression of Psl and alginate, this BAMP also exhibits immunogenic activity, as indicated by the increased levels of anti-alginate antibodies in pwCF harboring mucoid *P. aeruginosa* biofilm in their chronically infected lungs (28).

The enhanced response of PMNs stimulated by biofilms with simultaneous overexpression of Psl and alginate suggests that PMNs possess PRRs capable of detecting distinct patterns in the matrix produced by *P. aeruginosa* biofilm. This recognition may occur through two potential mechanisms: the engagement of a greater number of PRRs by the Psl- and alginate-rich biofilm, or the activation of PRRs that recognize specific molecular patterns generated by the simultaneous overexpression of Psl and alginate. Although the precise PRRs involved have not yet been identified, the kinetics of PMN responses indicate that distinct PRRs may be activated. A notable distinction in the response dynamics was observed, as the oxidative burst in PMNs reached its peak significantly faster when stimulated by Psl- and alginate-rich biofilms compared with wild-type biofilms (18), which are known not to produce alginate (29). This temporal difference suggests that the immune response to biofilms enriched with Psl and alginate follows a different signaling cascade than that triggered by wild-type biofilms, potentially implicating separate PRRs and downstream pathways. The first step of the oxidative burst in PMNs is the production of superoxide by the NADPH oxidase 2 (NOX2) (30, 31). NOX2 activity is directed by phosphorylations executed by several kinases, including PKC and mitogen-activated protein kinase (MAPK) (32, 33). These kinases may be activated by distinct signaling pathways initiated by PRRs after recognition of BAMPs.

Even though several of the matrix components in *P. aeruginosa* biofilm have been firmly identified and associated with biofilm formation (3, 4, 34), PRRs exclusively directed against the matrix components in *P. aeruginosa* biofilm remain to be identified. PMNs express a diverse array of PRRs (35), among which, C-type lectin receptors (CTLRs) and Toll-like receptors (TLRs) have been implicated in recognizing matrix EPS produced by *P. aeruginosa* biofilms. Within the group of CTLRs, the mannose receptor (MR-CTLD4-7) and Dectin-2 may bind to Psl and Pel EPS expressed by *P. aeruginosa* biofilm, while upregulation of Psl or Pel by planktonic *P. aeruginosa* was not recognized by MR-CTLD4-7 (36). Accordingly, the recognition of Psl and Pel by MR-CTLD4-7 may be biofilm associated, which supports the ability of Psl and Pel to act as BAMPs. Among the TLRs, TLR2 and TLR4 may recognize free mannuronic acid polymers derived from the alginate of a mucoid isolate of *P. aeruginosa* resulting in increased production of TNF-α (37).

In addition to polysaccharides, protein, and lipids, the biofilm matrix may contain extracellular DNA (eDNA) (38). By comparing the effects of DNase treatment of *P. aeruginosa* biofilm, the ability of PMNs to recognize biofilm-associated eDNA has become evident according to the stimulation of cytokine IL-8 and IL-1β release, upregulation of CD11b, CD18, and CD66b activation markers, and phagocytic uptake of bacteria by the PMNs (39). The stimulatory effect of biofilm-associated eDNA was additionally verified by the diminished and DNase-independent response of PMNs exposed to biofilms of *lasIrhlI* mutants (39), which are low in eDNA (40). Furthermore, the association of the stimulatory eDNA with biofilm could be validated by the low and DNase-independent response of PMNs exposed to planktonic *P. aeruginosa*. Accordingly, eDNA in *P. aeruginosa* biofilms qualifies as a BAMP.

The surface-bound nature of the PRRs involved in the recognition of biofilm-associated eDNA excludes the participation of TLR9 (39), which is expressed in endolysosomal compartments (41) and recognizes internalized bacterial DNA (42). Furthermore, since biofilm-associated eDNA did not influence the respiratory burst by PMNs (39), the PRRs recognizing biofilm-associated eDNA are unlikely to participate in the extra production of ROS induced by Psl and alginate in biofilms. However, identification of PRRs recognizing BAMPs awaits further investigation.

So far, we have provided examples of roles of biofilm matrix components as BAMPs. In addition, there is evidence that signaling molecules, such as acyl homoserine lactones (AHLs) and cyclic-di-GMP, can affect the innate immune system. The AHLs are quorum-sensing signal molecules, and they are expressed at high levels in biofilms (43), whereas cyclic-di-GMP is a biofilm regulator molecule known to be produced at high levels when bacteria form biofilm (44). Evidence was provided that the C12 AHL of *P. aeruginosa* can attenuate TLR4-dependent innate immune responses, e.g., of macrophages, thereby potentially promoting persistent infection (45). In the case of cyclic-di-GMP, it was found that the molecule can activate several innate immune cells, e.g., macrophages and monocytes (46). Subsequently, evidence was provided that cyclic-di-GMP is sensed by a cytosolic surveillance pathway (47), and that it stimulates the production of type I IFN in a STING-dependent manner (48). Cyclic-di-GMP is not secreted by the bacteria, but extracellular cyclic-di-GMP may originate from a subpopulation of lysed bacteria in the biofilm (49). Thus, the AHL quorum-sensing molecules and the cyclic-di-GMP biofilm regulator can be regarded as BAMPs, although the AHLs appear to have an attenuating role, and it cannot be excluded that high concentrations of AHLs also can occur in very dense planktonic populations.

BAMP-like properties may also extend to the structural modifications of LPS that are associated with biofilm formation of some bacterial species. For example, biofilm formation of *P. aeruginosa* is associated with structural modifications of LPS both at the level of lipid A and polysaccharide moieties, and human monocytes were found to release higher levels of TNF and IL-6 in response to LPS isolated from *P. aeruginosa* biofilm compared with LPS isolated from planktonic bacteria (50).

## BAMPs IN *SALMONELLA ENTERICA* SEROVAR TYPHIMURIUM BIOFILMS

*S*. Typhimurium is an enteric pathogen causing intestinal infections after ingestion of contaminated food or water (51). Biofilm formation is frequently among the virulence characteristics supporting persistent *S*. Typhimurium infection, and the extracellular matrix plays important roles (52).

In addition to immunostimulatory polysaccharides and DNA, the extracellular matrix of biofilms may also contain proteins with immunoenhancing properties. In *S*. Typhimurium biofilm, the main matrix proteins are curli fibers (53, 54), and the expression of curli fibers is biofilm-associated due to the dependence on increased levels of cyclic-di-GMP, which is a characteristic of biofilm formation in Gram-negative bacteria (44). Curli fibers have been shown to induce increased expression of pro-inflammatory cytokines and chemokines, including IL-6, IL-8, TNFα, type I interferons (IFNs) and IL17 by macrophages, dendritic cells, or T-cells (54–58). Moreover, TLR2 was identified as a major PRR for recognizing and responding to curli fibers (55, 59). Accordingly, curli fibers can be regarded as BAMPs. The innate immune response to curli fibers in *S*. Typhimurium biofilms may play a pivotal role in bridging the activation of the adaptive immune system, as described below.

Beyond amyloid curli, *S*. Typhimurium biofilms also contain eDNA, which binds to curli, forming insoluble and ultra-stable curli-eDNA complexes (58). Comparisons of macrophages with knockouts of TLR2 or TLR9 indicated that the curli part of the curli/DNA complex first activates TLR2 on the membrane, which is then internalized, transporting the curli/DNA complex into the endosome, where the DNA part of the curli/DNA complex subsequently activates the DNA receptor TLR9 (58). Accordingly, eDNA and curli both function as BAMPs in enteric biofilms. In this two-step engagement of TLR2 and TLR9 by the curli/DNA complex, curli and DNA serve immunostimulatory roles, while the DNA component additionally acts as an immunogen, leading to the activation of type I IFN responses and the production of anti-dsDNA antibodies. Since these antibodies can function as autoantibodies, and the activation of type I IFN responses by curli/DNA complexes can contribute to immune dysregulation, these complexes are believed to play a role in triggering autoimmune responses. Consequently, curli/DNA complexes have been proposed as a potential mechanism underlying the development of autoimmune diseases, such as systemic lupus erythematosus (58). This suggests that BAMPs may play a role in the pathogenesis of autoimmune conditions.

Curli is also a major amyloid component of *Escherichia coli* biofilms and is produced by both commensal and pathogenic strains in the gut (60), where it may function as a BAMP. Being structurally similar to human amyloids, curli and human amyloids engage shared PRRs, which may trigger inflammation (61). During dysbiosis, increased abundance of Proteobacteria and their biofilms may elevate curli exposure, potentially contributing to chronic gastrointestinal, autoimmune, and neurodegenerative conditions through sustained immune activation (60, 61).

In addition to stimulation of TLR receptors, curli fibers, as found in the extracellular matrixes of *S*. Typhimurium and *E. coli* biofilms, may activate the NLRP3 inflammasome, leading to the production of IL-1β in macrophages (62). Given the potent pro-inflammatory properties of IL-1β (63), the capacity of other BAMPs to differentially engage inflammasome pathways compared with their planktonic counterparts remains inadequately addressed and warrants further investigations.

## BAMPs IN *STAPHYLOCOCCUS EPIDERMIDIS* BIOFILMS

*S. epidermidis* is normally present on human skin but also appears frequently as the causative agent of implant-related infections. The bacteria form biofilm on the implants, resulting in persistent infections that give rise to a profound local inflammatory response. This is evidenced by studies such as Wagner et al. (64) who analyzed samples from the infectious sites of 18 patients with implant-related infections and found in all cases high numbers of leukocytes present in the tissue surrounding the implants. The infiltrates consisted predominantly of PMNs, which were highly activated as indicated by their surface receptor pattern. The infiltrated PMNs had a prolonged life span as seen by upregulation of CD14, which requires *de novo* protein synthesis. The data indicated that local activation of the PMNs occurred in the infected tissues. The antimicrobial compounds produced by PMNs are toxic to the tissue surrounding the implants, and if the activities of the PMNs are not limited, collateral damage will occur. In more benign infections, the activities of PMNs are limited by monocytes and macrophages that, after a few days, infiltrate the infected site to scavenge the activated PMNs. However, in the case of implant-related infections, monocytes and macrophages evidently do not infiltrate the infectious site, and the prolonged activities of the PMNs result in substantial damage of the tissue surrounding the implants (64).

Hänsch and colleagues (65) found that opsonization with IgG antibodies and activated complement C3, C3b, and C3bi is not required for *S. epidermidis* biofilms to activate PMNs. Since antibody and complement-mediated opsonization is strictly required for activation of PMNs by planktonic *S. epidermidis* cells (66), the researchers wondered how *S. epidermidis* biofilms activate PMNs. It was hypothesized that specific components of the *S. epidermidis* biofilm matrix are responsible for activating the immune cells. The major polysaccharide in *S. epidermidis* biofilms is N-acetyl (1–6) β-glucosamine (PNAG), also termed polysaccharide intercellular adhesin (PIA) (67). Besides, the *S. epidermidis* biofilm matrix generally contains eDNA (68) and several proteins (69). To identify potential immune cell-stimulating components of the *S. epidermidis* biofilm matrix, Hänsch and colleagues isolated matrix material from *S. epidermidis* biofilm, and subjected it to fractionation by size exclusion chromatography, and then investigated the ability of the different fractions to activate PMNs, seen as release of lactoferrin from the immune cells and up-regulation of their activation-associated adherence proteins CD11b, CD18, and CD66 (70). It was found that fractions containing molecules with a weight of 40 kD to 66 kD activated the PMNs (70). Digestion of the EPS with trypsin reduced its ability to activate PMNs, suggesting that the activating substance was a protein (71). Biotin-labeled EPS was found to bind to PMNs, suggesting the presence of surface receptors on the PMNs (71). To gain information on the putative receptors involved, it was investigated if receptor-inactivating antibodies could prevent EPS-induced activation of PMNs. An antibody to TLR4 was found to inhibit the EPS-induced up-regulation of CD11b, suggesting that the TLR4 recognizes a component of the *S. epidermidis* biofilm matrix. The EPS also stimulated the oxidative burst from PMNs, but the TLR4-inactivating antibody did not interfere with this, suggesting the existence of alternative receptors (71). Because prior work had shown that the bacterial protein GroEL binds to TLR4 (72), its presence in the EPS was investigated and confirmed using Western blotting. Moreover, the presence of high quantities of GroEL in the matrix of *S. epidermidis* biofilms was demonstrated by the use of fluorescent GroEL antibodies. Depletion of GroEL by immunoadsorption reduced the capacity of the EPS to induce upregulation of CD11b. In addition, commercially available recombinant GroEL protein was found to induce both up-regulation of CD11b/CD66b and the oxidative burst from the PMNs (71).

In a follow-up study, Dapunt et al. (73) provided evidence that the *S. epidermidis* biofilm matrix and GroEL also stimulates PMNs to undergo NET formation. NET is an acronym for neutrophil extracellular trap and the process, also termed NETosis, consists of a controlled release of the PMN chromatin, laced with modified histones and granular proteins. NETosis occurs in response to infections, and the NET DNA has an antimicrobial effect on planktonic bacteria (74) but likely not on biofilms since eDNA is known to stimulate biofilm formation (38, 68). Dapunt et al. (73) found that *S. epidermidis* biofilms stimulated NETosis whether or not the biofilms were opsonized with human serum. Moreover, recombinant GroEL was found to stimulate NETosis.

GroEL is normally considered a chaperone that functions in the bacterial cytoplasm to hinder heat shock-induced protein misfolding (75), and thus its appearance in the biofilm matrix is a so-called moonlighting activity. However, GroEL fulfills the criteria for being a biofilm-associated molecular pattern and therefore can be considered as a BAMP. Since GroEL is widely conserved among bacteria, its role as a BAMP might apply to biofilms formed by various bacteria in addition to *S. epidermidis*.

Besides PMNs, there is also evidence that the matrix of *S. epidermidis* biofilms can activate complement. Complement components are soluble PRRs and are normally activated via three routes, where the classic pathway is initiated by antibody-mediated recognition, the alternative pathway is antibody independent and is triggered directly by the infectious agent, and the lectin pathway is activated by the interaction between mannose-binding lectin or serum ficolins and microbial polysaccharides. Kristian et al. (76) provided evidence that complement is required to mediate PMN-killing of planktonic *S. epidermidis* cells, and that it is activated via the alternative pathway. However, *S. epidermidis* biofilms were found to activate more complement than planktonic cells, and it was speculated that this might occur through the lectin pathway, since mannose-binding lectin and serum ficolins have lectin activity for GlcNAc residues of the PIA polysaccharide (76). In a subsequent study, Fredheim et al. (77) demonstrated that PIA in the matrix of *S. epidermidis* biofilms is a strong activator of complement. The researchers cultivated biofilms of an *S. epidermidis* wild-type and a PIA-negative isogenic strain in glucose-enriched media. Under these conditions, the *S. epidermidis* wild type formed biofilm with PIA as the main matrix component, whereas the PIA-negative strain formed biofilm with protein and eDNA as the main matrix components. Using a whole-blood model, the researchers found that the PIA-dependent biofilms activated complement much more than the protein/eDNA-dependent biofilms. The results were corroborated by experiments with purified PIA that also showed strong activation of the complement system. The pattern of complement activation products indicated that PIA stimulated complement via both the classical and the alternative pathway (77).

Since PIA evidently has potent proinflammatory properties by activating the complement system, we suggest that it is regarded as a BAMP. At the same time, studies have shown that biofilm-embedded *S. epidermidis* is protected from PMN killing, and that PIA is an important factor for this immune evasion (76, 78). It appears, therefore, that PIA both promotes inflammation and protects the biofilm-embedded bacteria against the immune response.

## BAMPs IN *STAPHYLOCOCCUS AUREUS* BIOFILMS

*S. aureus* is part of the microbiota of about one third of the human population where it is found mainly in the anterior nares. However, it also appears as a versatile pathogen known for causing a wide range of infections, from mild skin conditions like boils and cellulitis to more severe diseases such as pneumonia, sepsis, osteomyelitis, endocarditis, and implant-related infections (79). Its pathogenicity is largely attributed to its ability to form biofilm and to produce a variety of virulence factors, including toxins and enzymes that help it invade tissues, evade the immune system, and damage host cells. *S. aureus* can produce biofilm matrix components, such as PIA, eDNA, and various proteins (80). Polysaccharides or protein/eDNAs are the main biofilm matrix components dependent on the strain and conditions (80). Biofilm formation helps *S. aureus* to overcome immune responses and antibiotic treatment, and the emergence of antibiotic-resistant strains, such as methicillin-resistant *S. aureus* (MRSA), has complicated treatment further.

It has been documented that *S. aureus* produces and releases Panton–Valentine leucocidin (PVL) and γ-hemolysin AB (HlgAB) specifically in the biofilm mode of growth, and that these toxins elicit NET formation from PMNs (81, 82). The initial observation was that PMNs treated with spent medium from *S. aureus* biofilms showed a large proportion of cells with membrane damage and DNA release (81). On the contrary, this was not the case when PMNs were treated with spent medium from planktonic *S. aureus* cultures (81). The researchers subsequently showed that biofilm-spent medium from *S. aureus agr* and *saeRS* mutants did not affect the PMNs, indicating that one or more of the Agr/SaeRS-regulated phagocyte-puncturing toxins PVL, LukAB, LukED, HlgAB, and HlgCB were responsible for the effect. By studying the effect of spent medium from *S. aureus* biofilms formed by various toxin mutants, the researchers could conclude that both PVL and HlgAB damaged the membrane of PMNs and made them release DNA.

Bhattacharya et al. (81) speculated that the observed DNA release was a result of NETosis, and to verify NET formation, PMNs were incubated with biofilm-spent media from wild-type *S. aureus* or PVL/HlgAB mutants, and subsequently staining with antibodies against citrullinated H3 histone was carried out. Citrullination is a hallmark of NETosis and involves peptidyl arginine deiminase activity that converts chromatin arginine to the non-inherited amino acid citrulline. In the case of the *S. aureus* wild type, ample citrullination was observed, but this was not observed for the toxin mutants. The researchers then investigated the effect of PVL, HlgAB, and PMN NET formation on *S. aureus* biofilms. When wild-type *S. aureus* biofilms were incubated with PMNs, they induced NET formation from the PMNs, but the biofilms persisted. In contrast, when *S. aureus* PVL/HlgAB mutant biofilms were incubated with PMNs, their biomass was reduced, presumably due to PMN phagocytosis. The results indicated that the leukocidins PVL and HlgAB induce NETosis and also play a role in the persistence of *S. aureus* biofilms by mediating killing of PMNs (81). The results were corroborated with a porcine model of chronic burn wound infection. Wounds infected with the *S. aureus* wild type contained aggregates with a high number of bacteria and pronounced citrullination, whereas wounds infected with an *S. aureus* toxin mutant contained lower numbers of bacteria and a low level of citrullination.

Since the PVL and HlgAB proteins are produced primarily when *S. aureus* is in the biofilm mode of growth, and since these proteins induce PMNs to undergo NETosis, we suggest that they are classified as BAMPs. However, the mechanistic details of how they affect the PMNs to undergo NETosis are not known.

In a subsequent study, Bhattacharya et al. (82) discovered some of the mechanisms that enable *S. aureus* biofilms to survive the antimicrobial activities of PMNs. NETs usually have antimicrobial properties (83), and the researchers wondered why they did not affect the wild-type biofilm bacteria. Moreover, after NETosis, the PMNs, although anucleate, were able to continue phagocytosis of biofilm-associated *S. aureus*, and the researchers wondered why this did not affect the wild-type biofilm bacteria. Bhattacharya et al. (82) provided evidence that two distinct virulence mechanisms converge to facilitate the survival of wild-type *S. aureus* biofilms. First, the leucocidin LukAB, produced by *S. aureus* in biofilms, was found to prevent phagocytosis by killing the PMNs. Second, the nuclease Nuc, produced by *S. aureus* in biofilms, facilitated local breakdown of NET DNA and the survival of trapped *S. aureus* bacteria.

Recently, evidence was provided that amyloid/DNA complexes may function as BAMPs in *S. aureus* biofilms. Grando et al. (84) provided evidence that phenol-soluble modulins (PSMs) in *S. aureus* biofilms form complexes with eDNA and induce autoimmunity via a process that depends on TLR2 and TLR9 (84). PSMs are quorum-sensing-regulated molecules that are produced at high levels in *S. aureus* biofilms (80). The PSMs play a role in the development and stabilization of staphylococcal biofilms but are also involved in biofilm dispersal (85, 86). *S. aureus* PSMs can form amyloid fibrillar structures that resemble curli, and like curli, the PSM fibrils can bind to eDNA in biofilms (87). The results of Grando et al. (84) suggest that PSM/DNA complexes, similar to curli/DNA complexes (56), can function as BAMPs.

## CONCLUSION

We have presented findings supporting the presence of BAMPs in biofilms formed by both Gram-negative and Gram-positive bacteria. This underscores the necessity of particular mechanisms triggered and executed by the innate immune system to neutralize infectious bacterial biofilms. Our immune system is thought to eliminate solitary bacteria on a daily basis, for example, after tooth brushing that results in transfer of bacteria into the bloodstream. Likewise, our immune system could routinely eliminate smaller biofilms and microbial aggregates. However, in some cases, the immune system fails to eradicate the biofilms, and the prolonged activation of it leads to collateral damage on the surrounding tissues. Considering that PRRs and PAMPs are multivalent, the expression of BAMPs at immunostimulatory and immunogenic levels is unique for biofilms. This distinct expression pattern sets biofilms apart from planktonic bacteria in immune system interactions. Recognizing the role of BAMPs in biofilm-driven activation of the innate immune response will expose a unique class of immune stimuli that are potentially highly relevant to chronic infections and persistent dysbioses. This research may fill important gaps in our knowledge of molecular recognition pathways of immune cells. The translation of these basic discoveries into diagnostics and therapies may enable prevention of the collateral damage caused by hyperreactive immune responses and will enable efficient combat of biofilm-mediated diseases, which remain a major challenge in medicine today.

## References

[1] Costerton JW, Stewart PS, Greenberg EP. 1999. Bacterial biofilms: a common cause of persistent infections. Science 284:1318–1322. doi:10.1126/science.284.5418.1318 10334980

[2] Høiby N, Ciofu O, Johansen HK, Song Z, Moser C, Jensen PØ, Molin S, Givskov M, Tolker-Nielsen T, Bjarnsholt T. 2011. The clinical impact of bacterial biofilms. Int J Oral Sci 3:55–65. doi:10.4248/IJOS11026 21485309 PMC3469878

[3] Flemming HC, Wingender J. 2010. The biofilm matrix. Nat Rev Microbiol 8:623–633. doi:10.1038/nrmicro2415 20676145

[4] Pamp SJ, Gjermansen M, Tolker-Nielsen T. 2007. The biofilm matrix: a sticky framework, p 37–69. In Kjelleberg S, Givskov M (ed), The biofilm mode of life: mechanisms and adaptations. Horizon Bioscience, Norfolk, U.K.

[5] Jensen PØ, Givskov M, Bjarnsholt T, Moser C. 2010. The immune system vs. Pseudomonas aeruginosa biofilms. FEMS Immunol Med Microbiol 59:292–305. doi:10.1111/j.1574-695X.2010.00706.x 20579098

[6] Moser C, Jensen PØ, Thomsen K, Kolpen M, Rybtke M, Lauland AS, Trøstrup H, Tolker-Nielsen T. 2021. Immune responses to Pseudomonas aeruginosa biofilm infections. Front Immunol 12:625597. doi:10.3389/fimmu.2021.625597 33692800 PMC7937708

[7] Jensen PØ, Kolpen M, Kragh KN, Kühl M. 2017. Microenvironmental characteristics and physiology of biofilms in chronic infections of CF patients are strongly affected by the host immune response. APMIS 125:276–288. doi:10.1111/apm.12668 28407427

[8] Ciofu O, Tolker-Nielsen T. 2019. Tolerance and resistance of Pseudomonas aeruginosa biofilms to antimicrobial agents-how P. aeruginosa can escape antibiotics. Front Microbiol 10:913. doi:10.3389/fmicb.2019.00913 31130925 PMC6509751

[9] Janeway CA Jr. 1989. Approaching the asymptote? Evolution and revolution in immunology. Cold Spring Harb Symp Quant Biol 54:1–13. doi:10.1101/SQB.1989.054.01.003 2700931

[10] Medzhitov R, Janeway CA Jr. 1997. Innate immunity: the virtues of a nonclonal system of recognition. Cell 91:295–298. doi:10.1016/s0092-8674(00)80412-2 9363937

[11] Ciofu O, Moser C, Jensen PØ, Høiby N. 2022. Tolerance and resistance of microbial biofilms. Nat Rev Microbiol 20:621–635. doi:10.1038/s41579-022-00682-4 35115704

[12] Tolker-Nielsen T. 2014. Pseudomonas aeruginosa biofilm infections: from molecular biofilm biology to new treatment possibilities. APMIS Suppl 138:1–51. doi:10.1111/apm.12335 25399808

[13] Mayer-Hamblett N, Aitken ML, Accurso FJ, Kronmal RA, Konstan MW, Burns JL, Sagel SD, Ramsey BW. 2007. Association between pulmonary function and sputum biomarkers in cystic fibrosis. Am J Respir Crit Care Med 175:822–828. doi:10.1164/rccm.200609-1354OC 17234902 PMC2720115

[14] Starosta V, Rietschel E, Paul K, Baumann U, Griese M. 2006. Oxidative changes of bronchoalveolar proteins in cystic fibrosis. Chest 129:431–437. doi:10.1378/chest.129.2.431 16478863

[15] Sagel SD, Wagner BD, Anthony MM, Emmett P, Zemanick ET. 2012. Sputum biomarkers of inflammation and lung function decline in children with cystic fibrosis. Am J Respir Crit Care Med 186:857–865. doi:10.1164/rccm.201203-0507OC 22904182 PMC3530222

[16] Jensen ET, Kharazmi A, Lam K, Costerton JW, Høiby N. 1990. Human polymorphonuclear leukocyte response to Pseudomonas aeruginosa grown in biofilms. Infect Immun 58:2383–2385. doi:10.1128/iai.58.7.2383-2385.1990 2114367 PMC258823

[17] Jensen ET, Kharazmi A, Høiby N, Costerton JW. 1992. Some bacterial parameters influencing the neutrophil oxidative burst response to Pseudomonas aeruginosa biofilms. APMIS 100:727–733. 1325804

[18] Rybtke M, Jensen PØ, Nielsen CH, Tolker-Nielsen T. 2020. The extracellular polysaccharide matrix of Pseudomonas aeruginosa biofilms is a determinant of polymorphonuclear leukocyte responses. Infect Immun 89:e00631-20. doi:10.1128/IAI.00631-20 33077623 PMC7927924

[19] Briheim G, Stendahl O, Dahlgren C. 1984. Intra- and extracellular events in luminol-dependent chemiluminescence of polymorphonuclear leukocytes. Infect Immun 45:1–5. doi:10.1128/iai.45.1.1-5.1984 6329953 PMC263244

[20] Freitas M, Lima JLFC, Fernandes E. 2009. Optical probes for detection and quantification of neutrophils’ oxidative burst. A review. Anal Chim Acta 649:8–23. doi:10.1016/j.aca.2009.06.063 19664458

[21] Jensen ET, Kharazmi A, Garred P, Kronborg G, Fomsgaard A, Mollnes TE, Høiby N. 1993. Complement activation by Pseudomonas aeruginosa biofilms. Microb Pathog 15:377–388. doi:10.1006/mpat.1993.1087 8015418

[22] Pestrak MJ, Chaney SB, Eggleston HC, Dellos-Nolan S, Dixit S, Mathew-Steiner SS, Roy S, Parsek MR, Sen CK, Wozniak DJ. 2018. Pseudomonas aeruginosa rugose small-colony variants evade host clearance, are hyper-inflammatory, and persist in multiple host environments. PLoS Pathog 14:e1006842. doi:10.1371/journal.ppat.1006842 29394295 PMC5812653

[23] Learn DB, Brestel EP, Seetharama S. 1987. Hypochlorite scavenging by Pseudomonas aeruginosa alginate. Infect Immun 55:1813–1818. doi:10.1128/iai.55.8.1813-1818.1987 3038752 PMC260606

[24] Pedersen SS, Kharazmi A, Espersen F, Høiby N. 1990. Pseudomonas aeruginosa alginate in cystic fibrosis sputum and the inflammatory response. Infect Immun 58:3363–3368. doi:10.1128/iai.58.10.3363-3368.1990 2401567 PMC313661

[25] Davies DG, Chakrabarty AM, Geesey GG. 1993. Exopolysaccharide production in biofilms: substratum activation of alginate gene expression by Pseudomonas aeruginosa. Appl Environ Microbiol 59:1181–1186. doi:10.1128/aem.59.4.1181-1186.1993 8476292 PMC202258

[26] Yang L, Hengzhuang W, Wu H, Damkiaer S, Jochumsen N, Song Z, Givskov M, Høiby N, Molin S. 2012. Polysaccharides serve as scaffold of biofilms formed by mucoid Pseudomonas aeruginosa. FEMS Immunol Med Microbiol 65:366–376. doi:10.1111/j.1574-695X.2012.00936.x 22309122

[27] Granton E, Brown L, Defaye M, Moazen P, Almblad H, Randall TE, Rich JD, Geppert A, Abdullah NS, Hassanabad MF, et al.. 2024. Biofilm exopolysaccharides alter sensory-neuron-mediated sickness during lung infection. Cell 187:1874–1888. doi:10.1016/j.cell.2024.03.001 38518773

[28] Pedersen SS, Høiby N, Espersen F, Koch C. 1992. Role of alginate in infection with mucoid Pseudomonas aeruginosa in cystic fibrosis. Thorax 47:6–13. doi:10.1136/thx.47.1.6 1539148 PMC463537

[29] Wozniak DJ, Wyckoff TJO, Starkey M, Keyser R, Azadi P, O’Toole GA, Parsek MR. 2003. Alginate is not a significant component of the extracellular polysaccharide matrix of PA14 and PAO1 Pseudomonas aeruginosa biofilms. Proc Natl Acad Sci USA 100:7907–7912. doi:10.1073/pnas.1231792100 12810959 PMC164686

[30] Babior BM, Kipnes RS, Curnutte JT. 1973. Biological defense mechanisms. The production by leukocytes of superoxide, a potential bactericidal agent. J Clin Invest 52:741–744. doi:10.1172/JCI107236 4346473 PMC302313

[31] Gabig TG, Babior BM. 1979. The O_2_^−^-forming oxidase responsible for the respiratory burst in human neutrophils. Properties of the solubilized enzyme. J Biol Chem 254:9070–9074. 479180

[32] el Benna J, Faust LP, Babior BM. 1994. The phosphorylation of the respiratory burst oxidase component p47phox during neutrophil activation. Phosphorylation of sites recognized by protein kinase C and by proline-directed kinases. J Biol Chem 269:23431–23436. 8089108

[33] Inanami O, Johnson JL, McAdara JK, Benna JE, Faust LR, Newburger PE, Babior BM. 1998. Activation of the leukocyte NADPH oxidase by phorbol ester requires the phosphorylation of p47PHOX on serine 303 or 304. J Biol Chem 273:9539–9543. doi:10.1074/jbc.273.16.9539 9545283

[34] Limoli DH, Jones CJ, Wozniak DJ. 2015. Bacterial extracellular polysaccharides in biofilm formation and function. Microbiol Spectr 3. doi:10.1128/microbiolspec.MB-0011-2014 PMC4657554 26185074

[35] Thomas CJ, Schroder K. 2013. Pattern recognition receptor function in neutrophils. Trends Immunol 34:317–328. doi:10.1016/j.it.2013.02.008 23540649

[36] Singh S, Almuhanna Y, Alshahrani MY, Lowman DW, Rice PJ, Gell C, Ma Z, Graves B, Jackson D, Lee K, Juarez R, Koranteng J, Muntaka S, Daniel AM, da Silva AC, Hussain F, Yilmaz G, Mastrotto F, Irie Y, Williams P, Williams DL, Cámara M, Martinez-Pomares L. 2021. Carbohydrates from Pseudomonas aeruginosa biofilms interact with immune C-type lectins and interfere with their receptor function. NPJ Biofilms Microbiomes 7:87. doi:10.1038/s41522-021-00257-w 34880222 PMC8655052

[37] Flo TH, Ryan L, Latz E, Takeuchi O, Monks BG, Lien E, Halaas Ø, Akira S, Skjåk-Braek G, Golenbock DT, Espevik T. 2002. Involvement of toll-like receptor (TLR) 2 and TLR4 in cell activation by mannuronic acid polymers. J Biol Chem 277:35489–35495. doi:10.1074/jbc.M201366200 12089142

[38] Whitchurch CB, Tolker-Nielsen T, Ragas PC, Mattick JS. 2002. Extracellular DNA required for bacterial biofilm formation. Science 295:1487. doi:10.1126/science.295.5559.1487 11859186

[39] Fuxman Bass JI, Russo DM, Gabelloni ML, Geffner JR, Giordano M, Catalano M, Zorreguieta Á, Trevani AS. 2010. Extracellular DNA: a major proinflammatory component of Pseudomonas aeruginosa biofilms. J Immunol 184:6386–6395. doi:10.4049/jimmunol.0901640 20421641

[40] Allesen-Holm M, Barken KB, Yang L, Klausen M, Webb JS, Kjelleberg S, Molin S, Givskov M, Tolker-Nielsen T. 2006. A characterization of DNA release in Pseudomonas aeruginosa cultures and biofilms. Mol Microbiol 59:1114–1128. doi:10.1111/j.1365-2958.2005.05008.x 16430688

[41] Latz E, Schoenemeyer A, Visintin A, Fitzgerald KA, Monks BG, Knetter CF, Lien E, Nilsen NJ, Espevik T, Golenbock DT. 2004. TLR9 signals after translocating from the ER to CpG DNA in the lysosome. Nat Immunol 5:190–198. doi:10.1038/ni1028 14716310

[42] Hemmi H, Takeuchi O, Kawai T, Kaisho T, Sato S, Sanjo H, Matsumoto M, Hoshino K, Wagner H, Takeda K, Akira S. 2000. A Toll-like receptor recognizes bacterial DNA. Nature 408:740–745. doi:10.1038/35047123 11130078

[43] Davies DG, Parsek MR, Pearson JP, Iglewski BH, Costerton JW, Greenberg EP. 1998. The involvement of cell-to-cell signals in the development of a bacterial biofilm. Science 280:295–298. doi:10.1126/science.280.5361.295 9535661

[44] Jenal U, Reinders A, Lori C. 2017. Cyclic di-GMP: second messenger extraordinaire. Nat Rev Microbiol 15:271–284. doi:10.1038/nrmicro.2016.190 28163311

[45] Kravchenko VV, Kaufmann GF, Mathison JC, Scott DA, Katz AZ, Grauer DC, Lehmann M, Meijler MM, Janda KD, Ulevitch RJ. 2008. Modulation of gene expression via disruption of NF-kappaB signaling by a bacterial small molecule. Science 321:259–263. doi:10.1126/science.1156499 18566250

[46] Karaolis DKR, Means TK, Yang D, Takahashi M, Yoshimura T, Muraille E, Philpott D, Schroeder JT, Hyodo M, Hayakawa Y, Talbot BG, Brouillette E, Malouin F. 2007. Bacterial c-di-GMP is an immunostimulatory molecule. J Immunol 178:2171–2181. doi:10.4049/jimmunol.178.4.2171 17277122

[47] McWhirter SM, Barbalat R, Monroe KM, Fontana MF, Hyodo M, Joncker NT, Ishii KJ, Akira S, Colonna M, Chen ZJ, Fitzgerald KA, Hayakawa Y, Vance RE. 2009. A host type I interferon response is induced by cytosolic sensing of the bacterial second messenger cyclic-di-GMP. J Exp Med 206:1899–1911. doi:10.1084/jem.20082874 19652017 PMC2737161

[48] Jin L, Hill KK, Filak H, Mogan J, Knowles H, Zhang B, Perraud AL, Cambier JC, Lenz LL. 2011. MPYS is required for IFN response factor 3 activation and type I IFN production in the response of cultured phagocytes to bacterial second messengers cyclic-di-AMP and cyclic-di-GMP. J Immunol 187:2595–2601. doi:10.4049/jimmunol.1100088 21813776 PMC3159690

[49] Webb JS, Thompson LS, James S, Charlton T, Tolker-Nielsen T, Koch B, Givskov M, Kjelleberg S. 2003. Cell death in Pseudomonas aeruginosa biofilm development. J Bacteriol 185:4585–4592. doi:10.1128/JB.185.15.4585-4592.2003 12867469 PMC165772

[50] Ciornei CD, Novikov A, Beloin C, Fitting C, Caroff M, Ghigo JM, Cavaillon JM, Adib-Conquy M. 2010. Biofilm-forming Pseudomonas aeruginosa bacteria undergo lipopolysaccharide structural modifications and induce enhanced inflammatory cytokine response in human monocytes. Innate Immun 16:288–301. doi:10.1177/1753425909341807 19710099

[51] Fàbrega A, Vila J. 2013. Salmonella enterica serovar Typhimurium skills to succeed in the host: virulence and regulation. Clin Microbiol Rev 26:308–341. doi:10.1128/CMR.00066-12 23554419 PMC3623383

[52] Austin JW, Sanders G, Kay WW, Collinson SK. 1998. Thin aggregative fimbriae enhance Salmonella enteritidis biofilm formation. FEMS Microbiol Lett 162:295–301. doi:10.1111/j.1574-6968.1998.tb13012.x 9627964

[53] Zogaj X, Bokranz W, Nimtz M, Römling U. 2003. Production of cellulose and curli fimbriae by members of the family Enterobacteriaceae isolated from the human gastrointestinal tract. Infect Immun 71:4151–4158. doi:10.1128/IAI.71.7.4151-4158.2003 12819107 PMC162016

[54] Gallo PM, Rapsinski GJ, Wilson RP, Oppong GO, Sriram U, Goulian M, Buttaro B, Caricchio R, Gallucci S, Tükel Ç. 2015. Amyloid-DNA composites of bacterial biofilms stimulate autoimmunity. Immunity 42:1171–1184. doi:10.1016/j.immuni.2015.06.002 26084027 PMC4500125

[55] Tükel C, Raffatellu M, Humphries AD, Wilson RP, Andrews-Polymenis HL, Gull T, Figueiredo JF, Wong MH, Michelsen KS, Akçelik M, Adams LG, Bäumler AJ. 2005. CsgA is a pathogen-associated molecular pattern of Salmonella enterica serotype Typhimurium that is recognized by Toll-like receptor 2. Mol Microbiol 58:289–304. doi:10.1111/j.1365-2958.2005.04825.x 16164566

[56] Tükel C, Nishimori JH, Wilson RP, Winter MG, Keestra AM, van Putten JPM, Bäumler AJ. 2010. Toll-like receptors 1 and 2 cooperatively mediate immune responses to curli, a common amyloid from enterobacterial biofilms. Cell Microbiol 12:1495–1505. doi:10.1111/j.1462-5822.2010.01485.x 20497180 PMC3869100

[57] Nishimori JH, Newman TN, Oppong GO, Rapsinski GJ, Yen J-H, Biesecker SG, Wilson RP, Butler BP, Winter MG, Tsolis RM, Ganea D, Tükel Ç. 2012. Microbial amyloids induce interleukin 17A (IL-17A) and IL-22 responses via Toll-like receptor 2 activation in the intestinal mucosa. Infect Immun 80:4398–4408. doi:10.1128/IAI.00911-12 23027540 PMC3497426

[58] Tursi SA, Lee EY, Medeiros NJ, Lee MH, Nicastro LK, Buttaro B, Gallucci S, Wilson RP, Wong GCL, Tükel Ç. 2017. Bacterial amyloid curli acts as a carrier for DNA to elicit an autoimmune response via TLR2 and TLR9. PLoS Pathog 13:e1006315. doi:10.1371/journal.ppat.1006315 28410407 PMC5406031

[59] Tükel C, Wilson RP, Nishimori JH, Pezeshki M, Chromy BA, Bäumler AJ. 2009. Responses to amyloids of microbial and host origin are mediated through toll-like receptor 2. Cell Host Microbe 6:45–53. doi:10.1016/j.chom.2009.05.020 19616765 PMC2745191

[60] Miller AL, Bessho S, Grando K, Tükel Ç. 2021. Microbiome or infections: amyloid-containing biofilms as a trigger for complex human diseases. Front Immunol 12:638867. doi:10.3389/fimmu.2021.638867 33717189 PMC7952436

[61] Inan S, Wilson RP, Tükel Ç. 2025. IUPHAR review: from gut to brain: the role of gut dysbiosis, bacterial amyloids, and metabolic disease in Alzheimer’s disease. Pharmacol Res 215:107693. doi:10.1016/j.phrs.2025.107693 40086611

[62] Rapsinski GJ, Wynosky-Dolfi MA, Oppong GO, Tursi SA, Wilson RP, Brodsky IE, Tükel Ç. 2015. Toll-like receptor 2 and NLRP3 cooperate to recognize a functional bacterial amyloid, curli. Infect Immun 83:693–701. doi:10.1128/IAI.02370-14 25422268 PMC4294241

[63] Lopez-Castejon G, Brough D. 2011. Understanding the mechanism of IL-1β secretion. Cytokine Growth Factor Rev 22:189–195. doi:10.1016/j.cytogfr.2011.10.001 22019906 PMC3714593

[64] Wagner C, Kondella K, Bernschneider T, Heppert V, Wentzensen A, Hänsch GM. 2003. Post-traumatic osteomyelitis: analysis of inflammatory cells recruited into the site of infection. Shock 20:503–510. doi:10.1097/01.shk.0000093542.78705.e3 14625473

[65] Stroh P, Günther F, Meyle E, Prior B, Wagner C, Hänsch GM. 2011. Host defence against Staphylococcus aureus biofilms by polymorphonuclear neutrophils: oxygen radical production but not phagocytosis depends on opsonisation with immunoglobulin G. Immunobiology 216:351–357. doi:10.1016/j.imbio.2010.07.009 20850891

[66] Kobayashi SD, Voyich JM, Burlak C, DeLeo FR. 2005. Neutrophils in the innate immune response. Arch Immunol Ther Exp (Warsz) 53:505–517. 16407783

[67] Mack D, Fischer W, Krokotsch A, Leopold K, Hartmann R, Egge H, Laufs R. 1996. The intercellular adhesin involved in biofilm accumulation of Staphylococcus epidermidis is a linear beta-1,6-linked glucosaminoglycan: purification and structural analysis. J Bacteriol 178:175–183. doi:10.1128/jb.178.1.175-183.1996 8550413 PMC177636

[68] Qin Z, Ou Y, Yang L, Zhu Y, Tolker-Nielsen T, Molin S, Qu D. 2007. Role of autolysin-mediated DNA release in biofilm formation of Staphylococcus epidermidis. Microbiology (Reading) 153:2083–2092. doi:10.1099/mic.0.2007/006031-0 17600053

[69] Karamanos NK, Syrokou A, Panagiotopoulou HS, Anastassiou ED, Dimitracopoulos G. 1997. The major 20-kDa polysaccharide of Staphylococcus epidermidis extracellular slime and its antibodies as powerful agents for detecting antibodies in blood serum and differentiating among slime-positive and -negative S. epidermidis and other staphylococci species. Arch Biochem Biophys 342:389–395. doi:10.1006/abbi.1997.0107 9186502

[70] Meyle E, Brenner-Weiss G, Obst U, Prior B, Hänsch GM. 2012. Immune defense against S. epidermidis biofilms: components of the extracellular polymeric substance activate distinct bactericidal mechanisms of phagocytic cells. Int J Artif Organs 35:700–712. doi:10.5301/ijao.5000151 23065886

[71] Maurer S, Fouchard P, Meyle E, Prior B, Hänsch GM, Dapunt U. 2015. Activation of neutrophils by the extracellular polymeric substance of S. epidermidis biofilms is mediated by the bacterial heat shock protein GroEL. J Biotechnol Biomater 5. doi:10.4172/2155-952X.1000176

[72] Argueta JGM, Shiota S, Yamaguchi N, Masuhiro Y, Hanazawa S. 2006. Induction of Porphyromonas gingivalis GroEL signaling via binding to Toll-like receptors 2 and 4. Oral Microbiol Immunol 21:245–251. doi:10.1111/j.1399-302X.2006.00286.x 16842509

[73] Dapunt U, Gaida MM, Meyle E, Prior B, Hänsch GM. 2016. Activation of phagocytic cells by Staphylococcus epidermidis biofilms: effects of extracellular matrix proteins and the bacterial stress protein GroEL on netosis and MRP-14 release. Pathog Dis 74:ftw035. doi:10.1093/femspd/ftw035 27109773 PMC5985485

[74] Brinkmann V, Zychlinsky A. 2012. Neutrophil extracellular traps: is immunity the second function of chromatin? J Cell Biol 198:773–783. doi:10.1083/jcb.201203170 22945932 PMC3432757

[75] Stan G, Lorimer GH, Thirumalai D. 2022. Friends in need: how chaperonins recognize and remodel proteins that require folding assistance. Front Mol Biosci 9:1071168. doi:10.3389/fmolb.2022.1071168 36479385 PMC9720267

[76] Kristian SA, Birkenstock TA, Sauder U, Mack D, Götz F, Landmann R. 2008. Biofilm formation induces C3a release and protects Staphylococcus epidermidis from IgG and complement deposition and from neutrophil-dependent killing. J Infect Dis 197:1028–1035. doi:10.1086/528992 18419540

[77] Fredheim EGA, Granslo HN, Flægstad T, Figenschau Y, Rohde H, Sadovskaya I, Mollnes TE, Klingenberg C. 2011. Staphylococcus epidermidis polysaccharide intercellular adhesin activates complement. FEMS Immunol Med Microbiol 63:269–280. doi:10.1111/j.1574-695X.2011.00854.x 22077230

[78] Vuong C, Voyich JM, Fischer ER, Braughton KR, Whitney AR, DeLeo FR, Otto M. 2004. Polysaccharide intercellular adhesin (PIA) protects Staphylococcus epidermidis against major components of the human innate immune system. Cell Microbiol 6:269–275. doi:10.1046/j.1462-5822.2004.00367.x 14764110

[79] Cheung GYC, Bae JS, Otto M. 2021. Pathogenicity and virulence of Staphylococcus aureus. Virulence 12:547–569. doi:10.1080/21505594.2021.1878688 33522395 PMC7872022

[80] Otto M. 2018. Staphylococcal biofilms. Microbiol Spectr 6. doi:10.1128/microbiolspec.gpp3-0023-2018 PMC6282163 30117414

[81] Bhattacharya M, Berends ETM, Chan R, Schwab E, Roy S, Sen CK, Torres VJ, Wozniak DJ. 2018. Staphylococcus aureus biofilms release leukocidins to elicit extracellular trap formation and evade neutrophil-mediated killing. Proc Natl Acad Sci USA 115:7416–7421. doi:10.1073/pnas.1721949115 29941565 PMC6048508

[82] Bhattacharya M, Berends ETM, Zheng X, Hill PJ, Chan R, Torres VJ, Wozniak DJ. 2020. Leukocidins and the nuclease nuc prevent neutrophil-mediated killing of Staphylococcus aureus biofilms. Infect Immun 88:e00372-20. doi:10.1128/IAI.00372-20 32719153 PMC7504955

[83] Liu Q, Chen R, Zhang Z, Sha Z, Wu H. 2025. Mechanisms and immune crosstalk of neutrophil extracellular traps in response to infection. mBio 16:e0018925. doi:10.1128/mbio.00189-25 40237474 PMC12077121

[84] Grando K, Nicastro LK, Tursi SA, De Anda J, Lee EY, Wong GCL, Tükel Ç. 2022. Phenol-soluble modulins from Staphylococcus aureus biofilms form complexes with DNA to drive autoimmunity. Front Cell Infect Microbiol 12:884065. doi:10.3389/fcimb.2022.884065 35646719 PMC9131096

[85] Schwartz K, Syed AK, Stephenson RE, Rickard AH, Boles BR. 2012. Functional amyloids composed of phenol soluble modulins stabilize Staphylococcus aureus biofilms. PLoS Pathog 8:e1002744. doi:10.1371/journal.ppat.1002744 22685403 PMC3369951

[86] Wang R, Khan BA, Cheung GYC, Bach T-HL, Jameson-Lee M, Kong K-F, Queck SY, Otto M. 2011. Staphylococcus epidermidis surfactant peptides promote biofilm maturation and dissemination of biofilm-associated infection in mice. J Clin Invest 121:238–248. doi:10.1172/JCI42520 21135501 PMC3007140

[87] Schwartz K, Ganesan M, Payne DE, Solomon MJ, Boles BR. 2016. Extracellular DNA facilitates the formation of functional amyloids in Staphylococcus aureus biofilms. Mol Microbiol 99:123–134. doi:10.1111/mmi.13219 26365835 PMC4715698

